# A Serious Game–Derived Index for Detecting Children With Heterogeneous Developmental Disabilities: Randomized Controlled Trial

**DOI:** 10.2196/14924

**Published:** 2019-10-24

**Authors:** Changbae Bang, Yelin Nam, Eun Jae Ko, Wooseong Lee, Byungjae Kim, Yejin Choi, Yu Rang Park

**Affiliations:** 1 Yonsei University College of Medicine Seoul Republic of Korea; 2 Department of Rehabilitation Medicine, University of Ulsan College of Medicine Ulsan Republic of Korea; 3 DoBrain Co, Ltd Seoul Republic of Korea; 4 Department of Biomedical Systems Informatics, Yonsei University College of Medicine Seoul Republic of Korea

**Keywords:** serious game, developmental disabilities, mobile game, cognitive screening tool, machine learning

## Abstract

**Background:**

Developmental disabilities are a set of heterogeneous delays or difficulties in one or more areas of neuropsychological development. Considering that childhood is an essential stage of brain development and developmental delays lead to personal or social burdens, the early detection of childhood developmental disabilities is important. However, early screening for developmental disabilities has been a challenge because of the fear of positive results, expensive tests, differences in diagnosis depending on examiners’ abilities, and difficulty in diagnosis arising from the need for long-term follow-up observation.

**Objective:**

This study aimed to assess the feasibility of using a serious game–derived index to identify heterogeneous developmental disabilities. This study also examines the correlation between the game-derived index and existing neuropsychological test results.

**Methods:**

The randomized controlled trial involved 48 children with either normal development or developmental disabilities. In this clinical trial, we used 19 features (6 from the Korean-Wechsler Preschool and Primary Scale of Intelligence, 8 from the Psychoeducational Profile Revised, 2 from the Bruininks-Oseretsky Test of Motor Proficiency, Second Edition, and 3 from the Pediatric Evaluation of Disability Inventory) from neuropsychological tests and 9 (7 game scores, path accuracy, and completion rate) from the serious game, DoBrain. The following analysis was conducted based on participants’ baseline information and neuropsychological test and game-derived index data for one week: (1) we compared the baseline information between the normal development and developmental disabilities groups; (2) then we measured the correlation between the game-derived index and the neuropsychological test scores for each group; and (3) we built a classifier based on the game-derived index with a Gaussian process method and then compared the area under the curve (AUC) with a model based on neuropsychological test results.

**Results:**

A total of 16 children (normal development=9; developmental disabilities=7) were analyzed after selection. Their developmental abilities were assessed before they started to play the serious games, and statistically significant differences were found in both groups. Specifically, the normal development group was more developed than the developmental disabilities group in terms of social function, gross motor function, full-scale IQ, and visual motor imitation, in that order. Similarly, the normal development group obtained a higher score on the game-derived index than the developmental disabilities group. In the correlation analysis between the game-derived index and the neuropsychological tests, the normal development group showed greater correlation with more variables than the developmental disabilities group. The game-derived index–based model had an AUC=0.9, a similar detection value as the neuropsychological test–based model’s AUC=0.86.

**Conclusions:**

A game-derived index based on serious games can detect children with heterogenous developmental disabilities. This suggests that serious games can be used as a potential screening tool for developmental disabilities.

**Trial Registration:**

Clinical Research Information Service KCT0003247; https://cris.nih.go.kr/cris/en/search/search_result_st01 .jsp?seq=12365

## Introduction

Developmental disabilities are among the most common diseases in children younger than 5 years old. Developmental disabilities are a set of heterogenous delays or difficulties in one or more developmental milestones, including learning, self-care, social interactions, and movement. The global number of children under 5 years old with developmental disabilities was 52.9 million in 2016, accounting for 13.3% of total years living with disability for these children. In 1990, 53.0 million children were living with developmental disabilities, indicating that there has not been much change since then [1]. In the United States, the prevalence of children diagnosed with developmental disabilities increased remarkably from 5.76% in 2014 to 6.99% in 2016 [2].

The brain is sensitive to stimulation during childhood, which is an essential stage of human development. It is the foundation for successive educational and vocational achievements, as well as society’s human capital development [3]. However, children with developmental disabilities are at a higher risk of substandard educational accomplishment, health status, and social relationships. More specifically, children with developmental disabilities have heightened difficulties reading, spelling, and counting due to shortages of phonological short-term memory or central, executive-loaded, working memory [4]. Additionally, developmental disabilities lead to sedentary lifestyles and seven times greater reported substandard emotional support in adulthood than in adults without disabilities. Consecutive developmental disabilities are risk factors of chronic health conditions such as high blood pressure, cardiovascular disease, diabetes, and chronic pain [5].

If a child fails to achieve a certain milestone, it is extremely hard to recover that milestone later in life. Therefore, late identification of developmental disabilities in children may require schools and families to pay for expensive programs [6]. Additionally, an understanding of the subsequent characteristics of a child’s developmental process requires a sensitive longitudinal examination by caregivers or specialists.

Given such a context, identifying developmental disabilities as early as possible has been deemed crucial. Multiple screening programs occurring in conjunction are recommended to detect early-stage developmental disabilities. For example, the American Academy of Pediatrics recommends frequent counseling, preventive care visits, and treatment visits for children [7]. Nevertheless, the early detection of developmental disabilities in children has not been performed adequately for many reasons, such as a lack of specific training for screening developmental disabilities, lack of time among specialists, fear of positive screen results, and failure to consider testing for developmental disabilities necessary [8]. In fact, even when early diagnosis and intervention programs are conducted in a timely manner, many instruments for diagnosis are administered in an environment distinct from an everyday setting. For example, children are directed to perform tasks in a laboratory setting or under the supervision of unfamiliar investigators.

To resolve these problems, approaches using serious games have been proposed for the early detection of developmental disabilities in children. Serious games are g*ames that do not have enjoyment, entertain[ment] or fun as their primary purpose* [9]*.*

Serious games targeted for health care use have seen a surge in use since 2004 [10]. Health care–targeted serious games are intended for health checks, disease detection, or rehabilitation. Some studies have been conducted that investigated games created for detecting various disorders, such as Parkinson disease, Alzheimer disease, or early dementia [11-14]. Other serious games have been targeted towards detecting developmental disabilities in children. For example, Anzulewicz created a machine learning model that can identify children with autism [15]. Alchalabi also studied ways to detect children with attention deficit hyperactivity disorder (ADHD) using a serious game integrated with electroencephalogram signals [16]. Additionally, many researchers have targeted adult cognitive impairment detection [17-21].

However, thus far there have been no studies about detecting general and heterogeneous developmental disabilities in children using a serious game, despite how important it is to detect these developmental disabilities. Referring children to specialists for clinical evaluation is also the most essential part of an early diagnosis of developmental disabilities. Therefore, this study aimed to develop a classifier that can distinguish between children with heterogeneous developmental disabilities and those with normal development using a game-derived index based on a serious game for improving cognitive ability. In this study, we collected gameplay data of children from Do Brain, a smart device-based serious game intended for the cognitive enhancement of children. 

## Methods

### Summary

This study was based on a single-blinded, parallel randomized controlled trial under the supervision of an independent data management team in the Asan Medical Center (Seoul, South Korea) The study consists of an intervention and a control group, with an allocation ratio of 1:1. Participants were openly recruited and enrolled in clinical studies via face-to-face evaluation by physicians and inspectors of neuropsychological testing. Children with normal development were 5-6 years old and were confirmed to have developed normally via examinations and specialists. Children with developmental disabilities were 5-7 years old but had a cognitive age of 4-6 years old. The recruitment period for this study was from October 2018 to January 2019. If the child was assigned to a group after the face-to-face evaluation, intervention was provided for 6 months. Our study only used the results obtained in the first week of the intervention.

Participants were too young to give consent, so we received “Subject explanatory note and consent forms” from the participants’ representatives (see Multimedia Appendix 1). The randomization, using a block size of four, was stratified depending on intervention and developmental disability. Randomization was done with the use of opaque, sealed envelopes. The statistician of the data management team generated the randomly allocated sequence with the use of the R program (R Core Team, Vienna, Austria). Physicians enrolled the patients and opened the envelope with the lowest available registration number within the appropriate stratum. Participants knew the group to which they belonged, but the physicians and the inspectors of the neuropsychological tests were blinded until the end of the trial.

The intervention of this study was based on a serious game and continued for 12 weeks for 40 minutes twice a week. The intervention was based on mobile serious gameplay at home. There were no human interventions other than early, functional, game usage training. To encourage sustainable use, we sent a text message to the representatives of participants who had not used it once in the preceding week. All clinical trial data were documented, coded, and stored on a computer at Asan Medical Center in Seoul, Korea.

In the current study, we wanted to explore the possibility of differentiating children with developmental disabilities from the normal population by analyzing gameplay patterns. We used the results obtained in the first week of the intervention because, as the intervention progressed, the cognitive function of the participants changed. This is because the cognitive function of children changes much faster than that of adults, and the game Do Brain was intended to enhance the cognitive function of children. The intervention was designed for a particular time and frequency of play (40 minutes twice a week).

**Figure 1:**
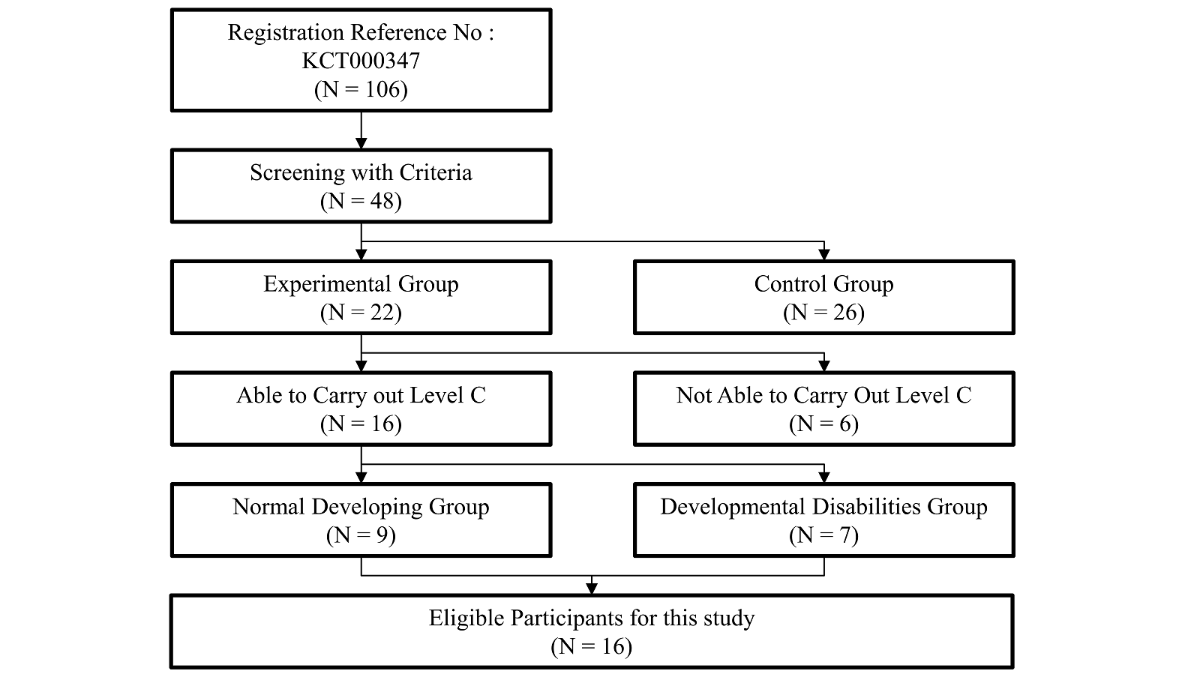
Participant selection flow chart.

Initially, 106 participants were recruited. The medical staff screened 48 children who met the recruitment criteria. Therefore, the study group consisted of 48 children with either normal development or developmental disabilities (Figure 1). Twenty-two of the children underwent intervention for 12 weeks (experimental group) and the other 26 participants were the control group. The control group that did not undergo intervention and children who could not carry out level C of the serious game were excluded from this study. The baseline information and data of 16 eligible participants during the first week of the intervention were analyzed. This study was approved by the Institutional Review Board of Asan Medical Center (IRB #2018-0989; Seoul, South Korea).

### Neuropsychological Tests

A set of neuropsychological tests was conducted to assess children’s developmental ability before the intervention period. This study employed the Korean-Wechsler Preschool and Primary Scale of Intelligence (K-WPPSI-IV) [22], a child’s intelligence test that provides a comprehensive assessment of overall intellectual capabilities. The target age of the test is from 1 year and 6 months to 7 years and 7 months. This study also used the Full-Scale IQ, which provides five basic indicators of intellectual function of a particular cognitive domain, including Verbal Comprehension Index (VCI), Visual Spatial Index (VSI), Fluid Reasoning Index (FRI), Working Memory Index (WMI), and the Processing Speed Index (PSI). In addition, the Psychoeducational Profile Revised (PEP-R) [23], which us used to assess the treatment capacity of children between 1 and 7.5 years old with autism and related developmental disabilities, was used to plan treatment programs. Additionally, the Bruininks-Oseretsky Test of Motor Proficiency, Second Edition (BOT-2) [24], which is administered in participants aged between 4 and 22 years, was used to check their motor development; it can also measure large and small muscle skills. The age range for the Pediatric Evaluation of Disability Inventory (PEDI) [25] is from 6 months to 7.5 years, and this test is used as a tool for evaluating independence in daily life through structured interviews with parents or caregivers.

### Serious Game

Do Brain is a serious game based on an animated cartoon. The game is a smart device–based application certified for child suitability from the iOS App store and the Google Play Store. It consists of games that require simple touch inputs, such as one-point touch, drag and drop, and rub. The object of the game is to enhance primary cognitive capacity (attention, orientation, memory), higher-level thinking abilities (problem solving, reasoning, concept formation), and meta-processing abilities (executive function, self-awareness). The application was downloaded to the personal smart device of each participant’s caregiver from the iOS App store or Google Play Store. The participants played the game in a natural environment, such as at home. A more detailed description is available at Do Brain homepage [26].

### Game-Derived Index

**Figure 2:**
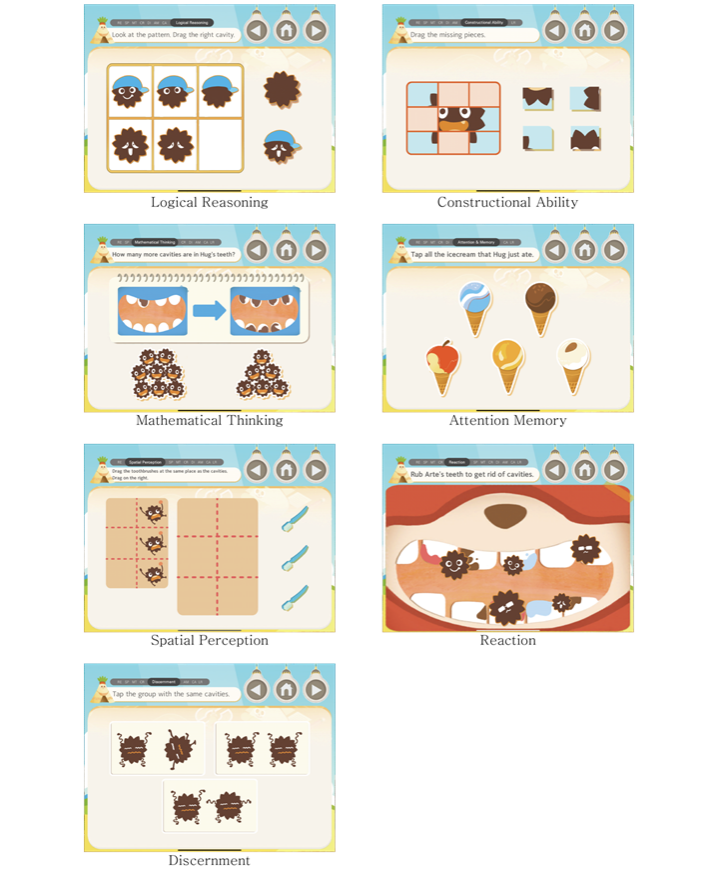
Screenshots of the serious game by game categories.

In the program, there are sections that each contain 6-8 games. Each game of a section tests for each of the categories of development, which includes: Spatial Perception, Mathematical Thinking, Attention Memory, Logical Reasoning, Constructional Ability, Discernment, and Reaction (Figure 2). Each game has 1-3 stages. In the first week of the intervention participants played through nine sections, which consisted of 61 games and 128 stages. For the first week of the intervention we computed the game score, path accuracy, and completion rate.

The game score was calculated using both the duration and the incorrect answer count. Duration referred to the time it took a participant to answer correctly, and incorrect answer count to the number of wrong answers/attempts. The game score increased as the participant completed a certain stage within a shorter time and with fewer incorrect answers. Therefore, the higher the score a participant obtained, the better they played. Below is the equation for the game score:

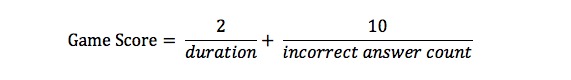



There were 16 drag-and-drop games in the 128 stages of the first week of the intervention, for which we computed path accuracy from both intended path and actual path. Intended path referred to the geometric distance between the start and end points and actual path to the distance of the finger’s movement. The path accuracy value ranged from 0 to 1, with values closer to 1 indicating that a participant moved their finger more precisely. Below is the equation for path accuracy:

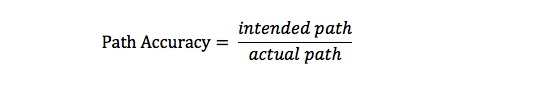



Meanwhile, completion rate was the ratio of the number of games played by each participant in a week divided by the quota for that week. It measured a participant’s compliance.

### Statistical Tests

In our study, we compared the baseline information between the normal development and developmental disabilities groups using the Mann-Whitney *U* test. We then measured the correlation between the game-derived index and a child’s neuropsychological test scores for each group using Pearson’s correlation method. Finally, we assessed the feasibility of using this serious game as a tool for detecting children with heterogeneous developmental disabilities. A classification model was built with a Gaussian process classifier, and model validation using leave-one-out cross validation (LOOCV) was conducted. LOOCV is a method for evaluating the prediction quality of a model built from a small dataset [27,28]. For the game-derived index–based model, we used nine features (7 game scores, path accuracy, and completion rate), and 19 features for the neuropsychological test–based model (6 K-WPPSI, 8 PEP-R, 2 BOT-2, 3 PEDI). In this study the multi-variate Gaussian process classifier of Rasmussen and Williams was used [29]. The classifier is based on Laplace approximation and makes predictions based on finite combinations of all random variables that have multivariate normal distributions. The receiver operating characteristic (ROC) and precision-recall (PR) curves were drawn from the validation results, and then the area under the curve (AUC) was calculated for each curve. Sensitivity, specificity, precision, true positive rate, and true negative rate were also measured. We set a significance level of 0.05. Data were processed and analyzed using R version 3.5.0 (R Core Team, Vienna, Austria), and Python 3.6 (Python Software Foundation, Wilmington, Delaware, United States; including the Pandas 0.22.0, NumPy 1.14.3, and Jupyter 1.0.0 packages).

## Results

### Participant Characteristics

#### Baseline Information

The normal development and developmental disabilities groups were comprised of nine and seven children, respectively. Chronological age was not significantly different between the two groups, but sex was significantly different in that the proportion of males in the developmental disabilities group was higher than in the normal development group. The K-WPPSI-IV test results, which measured a child’s cognitive ability, showed that full scale IQ was significantly different between the normal development and developmental disabilities groups (*P*=.008). Moreover, the normal development group surpassed the developmental disabilities group in all subcategories of K-WPPSI-IV. In particular, the normal development and developmental disabilities groups showed statistically significant differences in VCI (*P*=.01), VSI (*P*=.01), and FRI (*P*=.01; Table 1).

The developmental ages of the normal development and developmental disabilities groups were significantly different (*P*=.007). For all subcategories of PEP-R, the normal development group outperformed the developmental disabilities group. Specifically, statistically significant differences in Cognitive Verbal (*P*=.01), Cognitive Motion (*P*=.01), Fine Motor (*P*=.01), and Imitation (*P*=.03) categories were observed. Moreover, Visual Motor Imitation (*P*=.009) and Gross Motor (*P*=.005) showed more significant differences than the other categories (Table 1). For the data presented in Table 1, the Mann-Whitney *U* test was used as the statistical significance test.

**Table 1:** Comparison of the baseline information and neuropsychological tests of the normal development and developmental disability groups.

		Normal development group (n=9)	Developmental disability group (n=7)	*P* value
**Sex, n (%)**				
	Male	4 (44.4)	6 (85.7)	—^a^
	Female	5 (55.6)	1 (14.3)	—
Chronological age, mean (SD)		71.4 (6.20)	70.1 (8.01)	.35
**K-WPPSI-IV^b^, mean (SD)**				
	Full scale IQ	110.7 (18.3)	86.7 (13.7)	.008
	Verbal comprehension index	113.2 (16.7)	89.1 (15.7)	.02
	Visual spatial index	109.4 (13.8)	89.1 (19.0)	.01
	Fluid reasoning index	104.6 (11.6)	89.0 (13.7)	.01
	Working memory index	118.4 (16.8)	106.1 (15.5)	.14
	Processing speed index	99.3 (16.2)	88.1 (14.5)	.14
**PEP-R^c^, mean (SD)**				
	Developmental age	68.7 (5.0)	62.0 (5.4)	.007
	Cognitive verbal	73.6 (4.3)	64.4 (8.5)	.01
	Cognitive motion	70.0 (4.8)	63.4 (6.1)	.01
	Visual motor imitation	67.2 (6.2)	58.6 (5.4)	.009
	Gross motor	59.6 (0.9)	52.4 (11.7)	.005
	Fine motor	63.3 (4.2)	56.6 (5.4)	.01
	Perception	59.4 (2.9)	58.5 (2.9)	.07
	Imitation	63.1 (3.5)	60.6 (3.5)	.03
**BOT-2^d^, mean (SD)**				
	Fine motor	46.4 (19.9)	29.1 (13.8)	.05
	Manual coordination	30.2 (9.0)	22.3 (8.8)	.08
**PEDI^e^, mean (SD)**				
	Self-care	70.5 (1.5)	66.0 (5.6)	.10
	Movement	57.0 (3.7)	54.4 (5.6)	.20
	Social function	62.7 (2.5)	56.7 (4.6)	.005

^a^Not applicable.

^b^K-WPPSI-IV: Korean-Wechsler Preschool and Primary Scale of Intelligence.

^c^PEP-R: Psychoeducational Profile Revised.

^d^BOT-2: Bruininks-Oseretsky Test of Motor Proficiency, Second Edition.

^e^PEDI: Pediatric Evaluation of Disability Inventory.

#### Game-Derived Index

Overall, the normal development group showed better game scores than the developmental disabilities group on the game-derived index (Table 2). Logical Reasoning (*P*=.03), Constructional Ability (*P*=.04), Mathematical Thinking (*P*=.01), and Attention Memory (*P*=.04) were statistically significant in the two groups. In all subcategories of the game-derived index, the developmental disabilities group’s standard deviation was larger than in the normal development group. For the data presented in Table 2, the Mann-Whitney *U* test was used as the statistical significance test.

**Table 2:** Comparison of the game-derived index between the normal development and developmental disability groups.

Variable		Normal development group (n=9)	Developmental disability group (n=7)	*P* value
**Game score, mean (SD)**				
	Logical reasoning	8.28 (0.66)	7.16 (1.16)	.03
	Constructional ability	7.82 (0.69)	7.04 (1.01)	.04
	Mathematical thinking	9.12 (0.54)	8.13 (0.88)	.01
	Attention memory	9.20 (0.68)	8.50 (0.98)	.04
	Spatial perception	8.67 (0.58)	7.76 (0.97)	.06
	Reaction	8.31 (0.78)	7.89 (0.30)	.05
	Discernment	8.71 (0.55)	8.07 (1.07)	.19
Path accuracy, mean (SD)		0.795 (0.089)	0.750 (0.072)	.17
Completion rate, mean (SD)		0.991 (0.026)	0.910 (0.203)	.10

### Correlations Between the Neuropsychological Tests and Game-Derived Index

**Figure 3:**
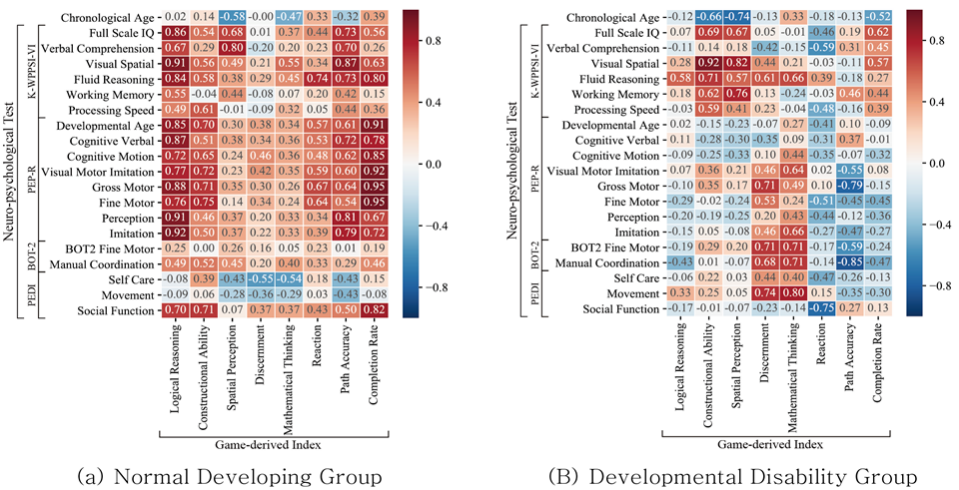
Correlation matrix for the game-derived index and neuropsychological tests of the normal development and developmental disability groups. K-WPPSI-IV: Korean-Wechsler Preschool and Primary Scale of Intelligence; PEP-R: Psychoeducational Profile Revised; BOT-2: Bruininks-Oseretsky Test of Motor Proficiency, Second Edition; PEDI: Pediatric Evaluation of Disability Inventory.

Correlations between the neuropsychological tests and game-derived index results were computed for each group using Pearson’s correlation method (Figure 3). Overall, the game-derived index and neuropsychological tests showed higher correlation values for the normal development group than the developmental disabilities group. Logical reassignment and completion rate in the game-derived index showed a high correlation with neuropsychological tests. In the developmental disabilities group, the constructional ability of the game-derived index showed the highest correlation with VSI (0.92). 

### Game-Derived Index– and Neuropsychological Test–Based Classifiers

**Figure 4:**
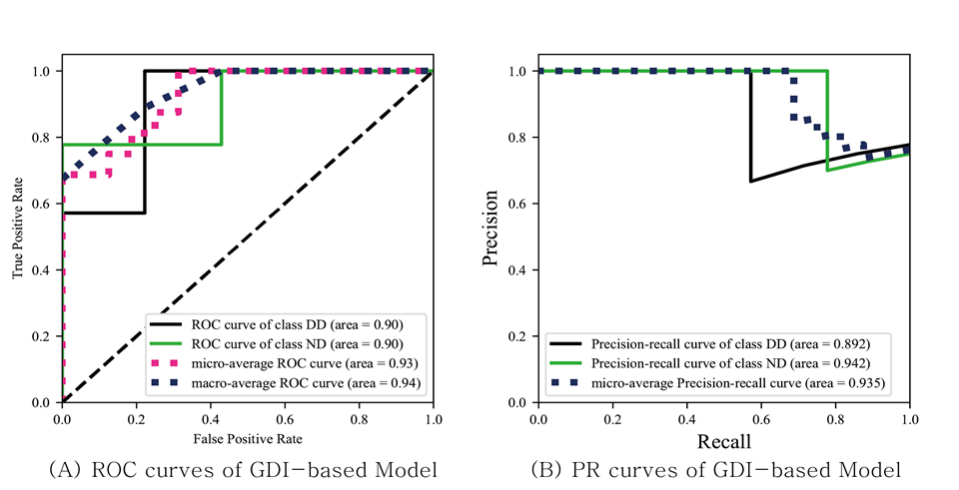
The receiver operating characteristic and precision-recall curves for leave-one-out cross validation of the model based on the game-derived index from the serious game. ROC: receiver operating characteristic; DD: developmental disabilities; ND: normal development; PR: precision recall; GDI: game-derived index.

**Figure 5:**
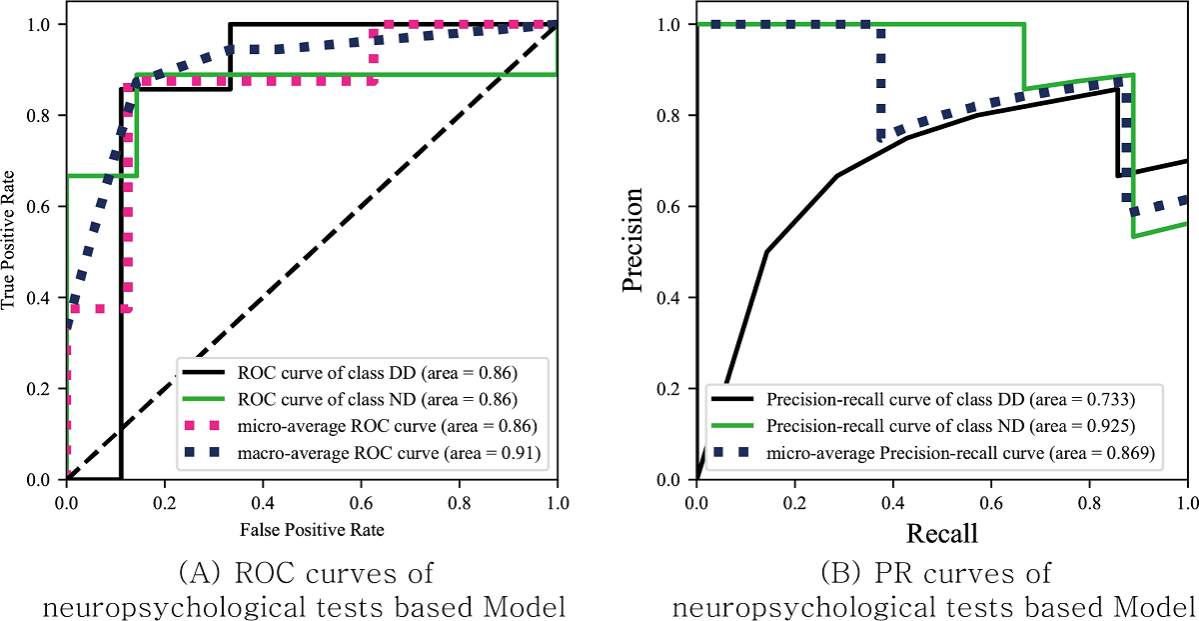
The receiver operating characteristic and precision-recall curves for leave-one-out cross-validation of the model based on the neuropsychological tests. ROC: receiver operating characteristic; DD: developmental disabilities; ND: normal development; PR: precision recall.

As shown in Figures 4 and 5, the game-derived index–based, classifier has a similar AUC to the neuropsychological test–based classifier. Sensitivity and specificity showed similar patterns. The game-derived index classifier had a sensitivity of 0.714 and a specificity of 0.778, whereas the neuropsychological test–based classifier had a sensitivity of 0.857 and a specificity of 0.778.

No serious adverse events or side effects were observed in the normal development and developmental disabilities groups.

## Discussion

### Principal Results

We built a classifier from the game-derived index that could distinguish children with developmental disabilities from those with normal development. In contrast to some models that can detect a specific type of disease, such as autism or ADHD, our classifier is the first model to detect children with heterogeneous types of developmental disabilities. This suggests that serious games have the potential for screening the general developmental disability status of children. Considering that the positive diagnosis of developmental disabilities should be conducted through a specialist’s exam, our classifier that detects general and heterogeneous developmental disabilities could be used to refer children with developmental disabilities to specialists for more specific and expert exams.

Additionally, we compared the game-derived index with the results of the neuropsychological tests to determine the characteristics of the normal development group that helped them obtain a better game-derived index than the developmental disabilities group. As many serious games that assess the cognitive function of humans lack psychometric analysis and comparisons with standardized studies [30], the comparison between the game-derived index and the neuropsychological tests in this study provides a more comprehensive interpretation of the serious gameplay of children.

### Differences in Game-Derived Index Between Normal Development and Developmental Disabilities Groups

Game score, path accuracy, and completion rate were computed to assess participants’ gameplay. Although some features of the game-derived index lacked statistical significance, the overall pattern showed that the normal development group played better than the developmental disabilities group.

Game score represents how fast and correctly a participant solved a problem. The serious game used in this study is based on a cognitive counseling program for children. As the normal development group showed higher intelligence scores on the neuropsychological tests, this means that they answered correctly with fewer attempts and in less time. For the normal development group, many game-derived index features showed a positive linear correlation with neuropsychological tests. More specifically, Logical Reasoning was correlated with Full Scale IQ and Developmental Age with coefficients of 0.86 and 0.85, respectively.

By contrast, most game-derived index features of the developmental disabilities group did not show linear correlations or had negative correlations. These results can be attributed to the following reasons. First, the developmental disabilities were heterogenous in the developmental disabilities group. Also, the developmental disabilities group had a small, restricted sample. Moreover, some studies indicate that cognitive assessment is difficult for patients suffering from neuropsychological disorders [31-33]. More specifically, when converting raw scores to standardized scores, performance variations that need to be detected often become obscure due to the flooring effect [34,35]. The flooring effect refers to a phenomenon in which the measuring tool cannot discriminate among those who belong to the lower level of the characteristic to be measured [36]. The flooring effect may occur when the score range itself is limited, or when inspection is too difficult. As standardized tests have such limitations, recent trends in intellectual disability research emphasizes narrating the cognitive signatures of conditions throughout their lifetime rather than depending on test scores [31]. Although many game-derived index features did not have positive correlations, constructional ability, spatial perception, and completion rate had correlation coefficients of 0.69, 0.67, and 0.62, respectively.

Path accuracy indicates fine motor function and visuospatial ability. According to Vatavu, children who are better developed in visuospatial processing have a high path accuracy score [37]. In our study, the normal development group’s path accuracy score was higher than the developmental disabilities group, but the difference was not statistically significant. Path accuracy scores were highly correlated with the VSI of the K-WPPSI in the normal development group (*r*=0.87), which shows a similar pattern to Vatavu’s findings. These findings indicate that children with better VSI scores are better at interpreting geometrical relationships. As a result, they understand drag-and-drop games better and can move their finger in a more intentional path. However, the normal development group’s path accuracy did not correlate well with Fine Motor index of PEP-R and BOT-2, mainly because testing of PEP-R or BOT-2 differs from a drag-and-drop game. Moreover, the developmental disabilities group’s path accuracy had negative correlation with the neuropsychological tests’ VSI and Fine Motor index. This negative correlation can be attributed to the developmental disabilities group’s heterogeneity and small sample size.

Completion rate is a measure of participant compliance. In this serious game, participants may skip a stage when they find it difficult. As can be observed in the game score results, the normal development group outperformed the developmental disabilities group in gameplay. Additionally, the normal development group’s mean completion rate was higher than the developmental disabilities group’s. Notably, the standard deviation differed greatly between the normal development (0.026) and developmental disabilities (0.203) groups. The large standard deviation of the developmental disabilities group’s completion rate is due to some developmental disabilities participants skipping many more stages than the others.

### Classification Model

The model built from the game-derived index performed adequately compared with that built from neuropsychological tests, showing the discriminating power of the game-derived index. This result suggests that serious games have the potential for detecting children with developmental disabilities. As children with heterogeneous developmental disabilities show complicated characteristics, characterizing them with high sensitivity is difficult. In contrast to models that detect specific developmental disorders developed in previous studies, the model built in this study is the first to detect general developmental disabilities.

### Comparison With Previous Studies

Some studies have been conducted regarding developmental delay detection with serious games. Previous works utilized more devices, required more resources, or could detect only specific diseases.

Alchalabi’s study that detected ADHD patients with an Electroencephalogram (EEG)-based serious game [16] required an EEG reader and a special gaming setting. However, our study only utilizes smart devices and is done in the home setting. Like our study’s use of neuropsychological tests for clinical diagnosis, Alchalabi’s study was also meaningful in that it used EEG with some diagnostic value.

Elhady build a speech disability detection game with limited speech resources [38]. Our study uses touch input from a smart device while it records the voice. However, they targeted a very specific speech disability population suffering from the phonemes /s/ and /r/. There is a need to cover broader speech disabilities, as there exist heterogenous speech disabilities [39].

Garcia and Ruiz created a children’s psychomotor delay screening process called Ubiquitous Detection Ecosystem to Care and Early Stimulation for Children with Developmental Disorders (EDUCARE) [40] based on a smart toy. In EDUCARE, the video that children played on the toy had been analyzed by developmental experts. However, EDUCARE only assessed motor function and depended on lengthy screening by experts. Our study overcomes this by assessing more developmental areas and immediately giving numerical developmental indices to caregivers. In a different respect, EDUCARE utilized methods more related to everyday objects, such as stackable cubes, than to our game-based approach.

### Limitations

Our study population is insufficient for generalizability of our findings to the general population. However, our findings show the feasibility of using this serious game to screen for general and heterogeneous developmental disabilities. As children continue to develop in various areas in the earlier stages of developmental disabilities, a definite diagnosis of the disease is difficult. Our model is useful because of the flexibility and heterogeneous characteristics of developmental disabilities. In the future, we will widen our study population to enhance the generalizability of our findings.

In a clinical situation, a neuropsychological test is essential for the diagnosis of developmental disabilities. Therefore, the estimation of neuropsychological test results with a game-derived index could provide caregivers and specialists with more information. However, our study did not build a model that estimates neuropsychological test results from the game-derived index because of the small sample size. Future studies must build a neuropsychological test estimation model more complex than our simple linear correlation model.

### Conclusion

The game-derived index patterns observed in serious gameplay in the normal development and developmental disabilities groups were different. In particular, the neuropsychological tests and game-derived index have significant differences in the correlations between groups. This result is a potential indicator that game-derived index can distinguish developmental disabilities from normal development, and the model based on it showed similar performance to neuropsychological tests that constitute conventional developmental ability tests. This suggests that serious games can be used as a potential screening tool for developmental disabilities.

## Appendix

Multimedia Appendix 1 Informed consent for the randomized controlled trial.

Multimedia Appendix 2 CONSORT‐EHEALTH checklist (V 1.6.1).

## Abbreviations

ADHD: attention deficit hyperactivity disorder
AUC: area under the curve
BOT-2: Bruininks-Oseretsky Test of Motor Proficiency, Second Edition
EDUCARE: Ubiquitous Detection Ecosystem to Care and Early Stimulation for Children with Developmental Disorders
EEG: electroencephalogram
FRI: Fluid Reasoning Index
K-WPPSI-IV: Korean-Wechsler Preschool and Primary Scale of Intelligence
LOOCV: leave-one-out cross validation
PEDI: Pediatric Evaluation of Disability Inventory
PEP-R: Psychoeducational Profile Revised
PR: precision recall
PSI: Processing Speed Index
ROC: receiver operating characteristic
VCI: Verbal Comprehension Index
VSI: Visual Spatial Index
WMI: Working Memory Index
