# Supplementary material for: A Serious Game–Derived Index for Detecting Children With Heterogeneous Developmental Disabilities: Randomized Controlled Trial
Source: JMIR Serious Games. 2019 Oct 24;7(4):e14924. doi: 10.2196/14924 (PMC6838693; doi:10.2196/14924)
Supplement: Multimedia Appendix 1 [file games_v7i4e14924_app1.docx]

| **Informed Consent Form** |
| --- |
| Research description   \| Title. \| The effect of DoBrain on cognition, language, and fine motor function in normal developing children and children with delayed development: single center, randomized, investigator-driven pilot study \| \| \| \| \| \| \| --- \| --- \| --- \| --- \| --- \| --- \| --- \| \| Director \| Name \| In Young Sung \| Institution \| Department of Physical Medicine and Rehabilitation \| Tel. \| +82-2-3010-3800 \| \| co-researcher \| Name \| Eun Jae Ko \| Institution \| Department of Physical Medicine and Rehabilitation, Ulsan University Hospital, University of Ulsan College of Medicine, Ulsan, South Korea \|  \| \| \| co-researcher \| Name \| Jin suk Yook \| Institution \| Department of Physical Medicine and Rehabilitation \| Tel. \| +82-2-3010-3783 \| \| Researh Manager \| Name \| Mi Jin Hong \| Institution \| Department of Physical Medicine and Rehabilitation \| Tel. \| +82-2-3010-1871 \|   This informed consent form is to help you understand the clinical study procedure, and contains detailed explanations on the study. Please read the document and after thorough consideration, decide whether to participate or not.  Only with your spontaneous consent, you will participate in the study. You can also decide not to participate  Therefore, it is critical that you understand the objective and procedure of the study, personnel participating in the study, benefits and risks involved in the tests before deciding whether to participate in the study or not.  Please read the following articles that are the answers to questions you may have about this research, and you can ask any questions as you read them.  You (or your legal guardian) must sign this consent form to begin participating in this study after you have heard the answers to all questions you are curious about and have decided that you would like to participate in this study.   The Research Director (or the research manager entrusted by the Research Director) who explained the study to you should also sign the consent form and state the date in his own handwriting.   Your signature means that you have heard and understood the procedures and risks of this study.   The participants in this are those who have difficulty understanding the contents of the consent form,and the consent of the child is legally impossible. So, the parent and legal guardian of the child are given responsibility for clinical participation.  Your signature on this document means your child wants to participate in this study.  Should I be involved in this research?  The purpose of this study is to find out the effect on cognition, language, and fine motor function of DoBrain in normal developing children and children with delayed. If your child participates in this study and if our team can find out the effectiveness of the new tool for children’s cognitive development, we believe that it would be possible to spread affordable and useful programs to more children who need interventions for their cognitive development.  What is the current standard treatment for children who have developmental disabilities?  Currently, standard treatments (cognitive, language and fine motor skills) for children who have developmental disabilities is rehabilitation therapy. This treatment method is known to be effective in cognitive, linguistic and fine motor skills enhancement, and there are no common side effects.  If the muscle of child patient is getting too stiff, he/she can be prescribed medication to relieve stiffness. Excessive administration of this drug can cause side effects such as drowsiness, sagging and respiratory depression.  What is DoBrain?  DoBrain is a mobile program developed to help children who are 36-72 months of cognitive age improve their cognition. There are over 9,600 contents developed by brain research teams with more than 15 years of clinical experience. There are many other benefits that are different from traditional computerized cognitive therapy programs. First, it can be used on any smart devices in home or hospitals. Also, the program proceeds with storytelling so that even the children who cannot read are able to use them easily. It has been proved to be suitable for children (all age) by Google Store and App Store  Who will participate in the study?  All the participants should meet the inclusion criteria as follows:  (1) Normal Children  (A) Inclusion Criteria   - Children aged from 5 to 6 years old, who will not change their private education program during the study - Children verified as normal development by the doctors - Children with agreement from their lawful guardian   (B) Exclusion Criteria   - Children who cannot use DoBrain due to blindness or poor vision, or any other underlying condition - Children who have performed K-WPPSI (Korean-Wechsler Preschool and Primary Scale of Intelligence-Fourth edition) within the last 6 months.   (2) Children with developmental disabilities  (A) Inclusion Criteria   - Children aged from 5 to 18 years old, whose cognitive age is from 5 to 6 years old - Children verified as developmental disabilities by the doctors - Children who will not change their private education, and treatment program during the study - Children with agreement from their lawful guardian   (B) Exclusion Criteria   - Children who cannot use DoBrain due to blindness or poor vision, or any other underlying condition - Children who have performed K-WPPSI (Korean-Wechsler Preschool and Primary Scale of Intelligence-Fourth edition) within the last 6 months.   .  How does it proceed when you participate in the research?  Currently, we plan to find the effectiveness of DoBrain on cognitive ability, linguistic ability, and fine motor skill by performing the study on 20 normal children and 40 intellectually disabled children. The participants will be verified as normal development or developmental disability by be tested with K-WPPSI (Korean-Wechsler Preschool and Primary Scale of Intelligence-Fourth edition).   After that, the participants are then divided into test group and control group through 1:1 randomization. The test group will be asked to complete a minimum of 40 minutes of DoBrain sessions a time, twice a week for 12 weeks under the guidance of their parents. The control group will not use DoBrain.  Randomization ensures that all subjects have the same probability of being assigned to the test group and the same probability of being assigned to the control group to eliminate the bias that may occur from the discretion of the researcher.  During the treatment period, all patients receive their standard TAU provided by their usual care providers.  Participants in each groups are tested with Pediatric Evaluation of Disability Inventory (PEDI), Goal Attainment Scale (GAS), Bruininks-Oseretsky Test of Motor Proficiency-2 (BOT-2), Korea-Child Behavior Checklist (K-CBCL), Korean Wechsler Primary and Preschool Scale Intelligence-IV and Psycheducational Profile Revised (PEP-R) and before and after doing DoBrain intervention. The legal guardians will conduct the questionnaire, quality of the life for parent, which will take about three minutes before and after doing DoBrain intervention.  Only the legal Guardians of the test group are asked to do an assessment of satisfaction and side effects after the DoBrain programs through a questionnaire, which will take about three minutes. Whether the participants have completed the DoBrain sessions will be confirmed online.  The evaluation takes place in two institutions.   1. Seoul ASAN Medical Center 2. Pediatric Evaluation of Disability Inventory (PEDI) 3. Goal Attainment Scale (GAS) 4. Bruininks-Oseretsky Test of Motor Proficiency-2 (BOT-2) 5. Korea-Child Behavior Checklist (K-CBCL) 6. Ewha Child Counseling Center(Korean Ministry of Health and Welfare Certified Institute) 7. Korean Wechsler Primary and Preschool Scale Intelligence-IV 8. Psycheducational Profile Revised (PEP-R) 9. Quality of the life for parent 10. Survey on satisfaction of DoBrain   This experiment is a randomized single-blinded trial, meaning that the health care providers (doctors or nurses) will not be aware of the treatment allocation and only the participants themselves will be aware of the treatment allocation.  What should I do?  Once you decide to participate in the study and sign the consent form with your own handwriting, the study begins.  Children who participate in the study have cognitive, language, and dine motors functional assessment, development and behavior assessment, and social response and emotional behavior assessment before and after the intervention. The legal guardians will conduct the questionnaire, quality of the life for parent, which will take about three minutes before and after doing DoBrain intervention.  What are the possible side effects, risks and inconveniences?  DoBrain is mobile program that is designed to maximize the therapeutic and developmental effects of children, so excessive exposure to preschoolers or school-aged children with weak self-control may lead to smart device addiction. Side effects of smart device addiction are known as impaired vision, avoidance of interpersonal relationships, anxiety during withdrawal, and anxiety. Therefore, we ask guardians to limit the duration of a Dobrain less than one hour a day.  Although it is unlikely, if serious side effects occur and this seems to be closely related to DoBrain sessions, the legal guardians should report to the researchers and they will get the necessary action (e.g. health screenings, care in hospital, etc.) immediately. In addition, if you experience any other inconvenience, please consult with the researchers at any time.  What are the benefits of the study?  You will receive 20 weeks amount of Dobrain cognitive development software, and receive cognitive assessment before and after the test for free. Your participation will contribute to developing cognitive development software and improve lives of children with intellectual disability.  Do I have to pay for participating in the research?  All cost related to the test will be provided by the research team, and no separate expenses are required.  Participation Spontaneity  You can withdraw your consent to participate any time you want, and in this case study will be over and researcher will stop collecting study data from you. If you want your data to be discarded you should contact lead researcher and express your demand  Your decision will not affect your future treatment from the institution. You can decide not to participate, and it will not affect any of your treatment or result in any disadvantage in the future  Is there anything that I need to do when I’m involved in the research?  In the cases of the following, lead researcher might exclude you from further clinical protocol.  a. Study subject, or his legal guardian has asked to stop the test  b. Significant side effect has been found from the study  c. Child refuses to use, or due to his aggravated condition he cannot use Dobrain  d. After the study has started, subject has been to not satisfy the study criteria  e. Subject does not use Dobrain (2 weeks absence)  f. Abuse or misuse of Dobrain  g. Apply Dobrain software to other people  h. Medical staffs have decided that there is a serious medical condition that the study cannot continue  i. Surgery, drug treatment or any other medical care that might alter the result of the study is required during the study  j. Subject disobeys researchers’ instruction or does not follow the guidelines on the consent form and affects the study protocol’s credibility  k. Cannot observe subject due to his absence  l. Principal investigator decides that there is a significant problem with the study process  I’m curious about the confidentiality of my personal data.  If you agree to participate in the clinical study, you data such as your child's age and gender, date of birth, diagnosis, etc., will be kept on the computer. This information will be shared by researchers participating in the study and will be managed using passwords so that only the authorized researchers will be able to see it. However, our institution's clinical research review committee, clinical research protection center, and government agency representatives can review to confirm the correct progress of the study. If your child stops to participate in the study, your child's information collected prior to suspension can be used for research. All information obtained in this study will be processed in accordance with the laws of the Republic of Korea. Data collected from the study will be discarded after 3 years of storage after the end of research. Also, even if the results of your clinical studies are published, your child's identity will be kept confidential. By signing this agreement, you agree with all the above.  If there is new information about developmental disabilities while participating in the study, can you tell me about it?  While participating in the study, you will be informed about new treatments or information related to your child's developmental disabilities  Who should I contact if I have a question during the study or if there is a damage or problem with the study? If you have any furtherr questions on the study, or face any problem during the study please contact;  Research manager – name: Mi jin Hong Tel: 02-3010-1871 (24 hours available)    If you have any questions about welfare and rights as a participant in a clinical study while participating in a clinical study, or if you would like to talk to someone who is not directly involved in the study, please contact:  Clinical Research Protection Center  02-3010-7161  clinical trial committee of Asan Medical Center in Seoul 02-3010-7166 |
| **Informed Consent Form** |
| The effect of DoBrain on cognition, language, and fine motor function in normal developing children and children with delayed development: single center, randomized, investigator-driven pilot study  Before signing the agreement, please check the following items again and mark it in the box.   \| 1 \| Do you know that this clinical study is performed for research purposes? \| □ \| \| --- \| --- \| --- \| \| 2 \| Do you know enough about the purpose and method of this clinical study? \| □ \| \| 3 \| If you do not participate in this clinical study, you will not be disadvantaged. Also you can refuse or suspend your participation at any time if you participate. Did you know that there are no disadvantages? \| □ \| \| 4 \| Do you know that even if you do not participate in this clinical study, you still receive standard treatments known to date? \| □ \| \| 5 \| Do you know the benefits expected from participating in this clinical study? \| □ \| \| 6 \| Are you aware of the anticipated side effects and risks of participating in this clinical study? \| □ \|   I have listened to and understood all the explanations related to this clinical study, and I have received enough answers to all my questions. After I have had enough time, I voluntarily agree to participate in the study. I also allow my health information to be used and shared. I understand that I will receive a copy of my consent.    Name of Legal Representative ___________________________  Signature of Legal Representative _________________________  Date: Year_________Month ______Day______ Relationship _____________________  I confirm that I have adequately explained the clinical study to the participant of the study or to the subject of the study.  Name of Researcher_________________________  Signature of Researcher  Date: Year_________Month ______Day______   \| Sign the following only if applicable. \| \| --- \| \| This representative (parental guardian or spouse, etc.) agrees to participate in the clinical study on behalf of the participant. This is because participant is not able to express their opinions because of their lack of ability to express themselves.  Name of Legal Representative ___________________________  Signature of Legal Representative _________________________  The relationship between the participant _________________________  Date: Year_________Month ______Day______ \| \| I understand that the subject is not able to read consent form and other documented information and that the physician has fully explained about the study to the participant (or representative) and that the participant (or representative) (If available, a self-signed signature).  Name of Fair Observer 서명 ___________________________  Signature of Fair Observer _________________________    Date: Year_________Month ______Day______ \| |
